# Breaking barriers: supporting hematopoietic stem cell transplant program through collaborative radiation therapy service from a physically distant center

**DOI:** 10.1186/s43046-024-00221-7

**Published:** 2024-05-20

**Authors:** Subhas Pandit, Simit Sapkota, Abish Adhikari, Prakriti Karki, Roshani Shrestha, Deepak Suman Jha, Rajan Prajapati, Kanchan Sarga Nyaichyai, Bishesh Sharma Poudyal, Bishal Poudel, Anjani Kumar Jha

**Affiliations:** 1 Department of Clinical Oncology, Kathmandu Cancer Center, Tathali, Bhaktapur Nepal; 2 Department of Radiation Oncology, Kathmandu Cancer Center, Tathali Bhaktapur, Nepal; 3 Department of Research, Kathmandu Cancer Center, Tathali, Bhaktapur Nepal; 4 Clinical Hematology and Bone Marrow Transplant Unit, Civil Service Hospital, Minbhawan, Kathmandu, Nepal; 5 Medical Oncology Unit, Tribhuvan University Teaching Hospital, Maharajgunj, Kathmandu, Nepal

**Keywords:** Low-dose total body irradiation, Hematopoietic stem cell transplant, Graft-versus-host disease, Conditioning regimen, Low- and middle-income country

## Abstract

**Background:**

Total body irradiation (TBI) for hematopoietic stem cell transplant (HSCT) has certain distinct advantages, such as uniform dose distribution and lack of drug resistance, but it is not widely available in resource-constrained settings. To overcome the limitations of in-house radiotherapy services in hematology centers, we evaluated the feasibility of conducting HSCT programs in coordination with two physically distant centers using a reduced-intensity TBI protocol.

**Methods:**

Thirty-two patients with a median age of 20.5 years were included in the study. Fifteen patients were diagnosed with aplastic anemia, 10 patients with acute myeloid leukemia (AML), 3 patients with acute lymphocytic leukemia (ALL), and 4 patients with other hematological conditions. Conditioning regimens used were fludarabine plus cyclophosphamide in 29 cases, fludarabine-cytarabine ATG in 2 cases, and busulfan plus fludarabine in 1 case. The TBI dose was 3 Gy in 28 cases and 2 Gy in 4 cases. Patients were followed monthly after TBI, and the major toxicities were recorded.

**Results:**

The median follow-up was 22 months. The most common acute complication was acute graft-versus-host disease (GVHD), which occurred in 15.6% of patients. The major late complications were chronic GVHD (9.3%), Cytomegalovirus (CMV) infection (34.3%), and CMV-induced secondary graft failure (6.2%). Seventy-five percent of patients were alive, 21.9% were dead, and 1 patient was lost to follow-up.

**Conclusions:**

HSCT based on TBI is feasible even if the center lacks a radiotherapy facility by coordinating with a remote radiotherapy facility. without compromising the patient's outcome.

## Introduction

Hematopoietic stem cell transplantation (HSCT) is deemed to treat a variety of malignant or nonmalignant hematological disorders [1]. However, locating a suitable donor for allogenic transplantation, managing complications related to graft-versus-host disease (GVHD), and adopting the appropriate conditioning regimen before transplantation are still major challenges in low-resource settings [2]. The problem associated with the availability of human leukocyte antigens (HLA) from from matched siblings or matched unrelated donors for allogeneic transplants is partly overcome by the presence of haploidentical donors and considered a standard option in developing countries with limited resources due to their reduced financial resources [3].

The approach to the conditioning regimen of HSCT varies between transplant centers. Some of the commonly used conditioning regimens are categorized as myeloablative, reduced-toxicity, reduced-intensity, and non-myeloablative regimens, and these comprise either solely chemotherapy regimens or a combination of both chemotherapy and radiation [4].

Total body irradiation (TBI) can provide certain advantages over combination chemotherapy-based regimens, including uniform dose distribution throughout the body, avoidance of potential sanctuary sites, and reduced chemotherapeutic exposure [5]. The FORUM trial, a large international study in pediatric patients with high-risk acute lymphoblastic leukemia (ALL), demonstrated that myeloablative TBI plus etoposide prior to HSCT results in significantly improved overall survival, lower relapse incidence, and lower treatment-related mortality compared to myeloablative chemotherapy regimens, providing robust evidence of the use of TBI in the conditioning regimen for HSCT [6].

Low-dose TBI is commonly incorporated into haploidentical transplant settings. In low- and middle-income countries (LMIC) like Nepal, TBI-based protocols are much cheaper than chemotherapy-only regimens if the center is well equipped with radiotherapy equipment. In addition, the lack of essential chemotherapy drugs like busulfan, thiotepa, and treosulfan would make TBI-based regimens more attractive options for haploidentical transplants in LMIC. Moreover, fludarabine and cyclophosphamide are frequently used with doses as low as 2 Gy to successfully achieve engraftment in both pediatric and adult patients [4].

The lack of radiotherapy services is a reality in many LMICs [7]. Ideally, facilities for radiotherapy and stem cell transplantation should be located in the same hospital. However, in resource-limited settings, this scenario might not always be possible. This study investigated the feasibility of coordinating HSCT programs with two physically distant centers using a reduced-intensity TBI protocol to overcome the limitations of in-house radiotherapy services. The second aim of this study is to assess and compare complications associated with the reduced-intensity TBI protocol with HSCT programs featuring in-house radiotherapy facilities.

## Methods

### Study patients

Between November 2017 and November 2021, 32 consecutive patients who received TBI and underwent HSCT were included in the study. HSCT was started in the Civil Service Hospital of Nepal in 2016. However, TBI was not used for conditioning due to a lack of radiotherapy facilities. In 2017, to start a TBI-based conditioning regimen, a proposal was made to do TBI at the Kathmandu Cancer Center, which is located in a suburb of Kathmandu Valley at a distance of 15 km from the Civil Service Hospital.

The main challenge of this program was to use resources from two different institutions. To coordinate treatment plans and patient transfers, a group was created using instant messaging software (Viber) that included hematologists, residents, transplant nurses, radiation oncologists, physicists, and treating technicians. A focal person was identified at both the hematology center and the radiotherapy center. Once the patient was scheduled for HSCT, s/he visited the radiation center for registration and any necessary dosimetry assessments. The patient was then admitted to the hematology center and started conditioning chemotherapy. Once a patient was started on chemotherapy, it was communicated through a Viber group, and the focal person was informed about the scheduled day of TBI.

Post-TBI, stem cell therapy with peripheral blood stem cells was administered the following day. High-dose cyclophosphamide was used as GVHD prophylaxis after transplantation.

### Pre-TBI evaluation

Patients were called to visit the radiotherapy department to talk and be trained on how to pose during treatment. The patients’ information, such as name, sex, age, relevant medical diagnosis, and treatment schedule, was collected. Anthropometric measurements, including lateral separation (shoulder-to-shoulder distance), height, and weight of the patients were used to calculate the radiation dose.

### TBI delivery technique

In order to avoid office-hour traffic during patient transport, TBI was taken as the first case of the morning**.** Since registration was completed during the previous visits, the patient was immediately transferred from the ambulance to the radiotherapy vault after confirming their identity. For hygienic precautions, the radiotherapy vault was fumigated the night before treatment. All involved personnel used masks and gloves, and the treatment couch was disinfected before treatment. Standard hospital hygiene measures, including hand disinfection, were strictly followed.

**Fig. 1:**
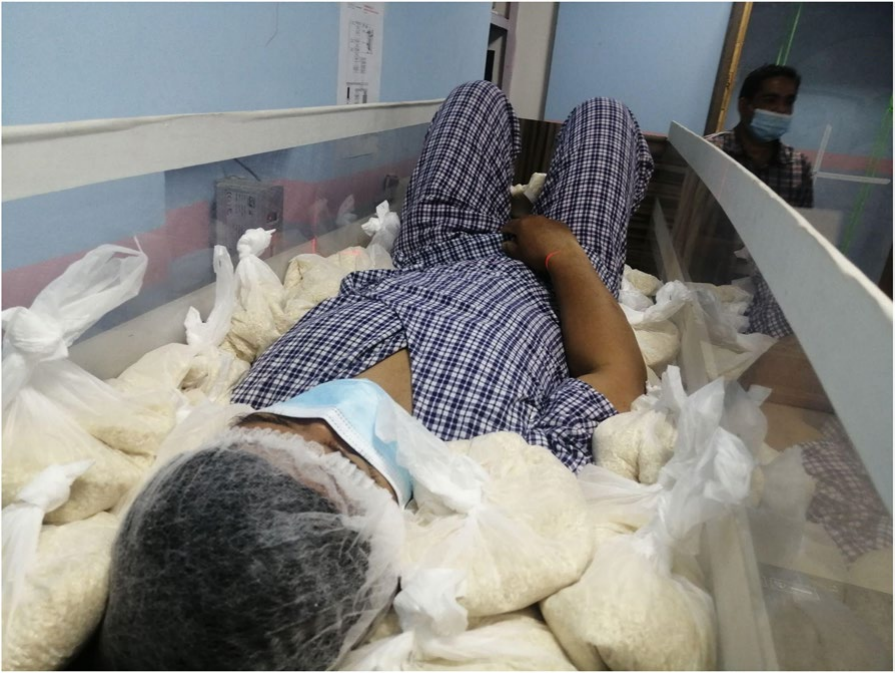
Patient positioning in customized treatment couch for TBI. Rice bags are placed on both sides which act as missing tissue compensators

Patients were positioned in a supine position at an extended source-to-skin distance (SSD) of 330 cm on a custom-made wooden couch with a lucite sheet of 1 cm on both sides. The couch was kept on top of the trolley to facilitate changing its direction. The treatment involved stationary beams with field sizes of the order of 70 × 200 cm^2^ encompassing the whole length of the patient. The collimator was rotated to 45° to get the field size, equal to the diagonal length of the square field size hence accommodating the longest possible side. Patients were kept at central, 75% of the light field size, which was considered a dosimetric field size to account for clipping of the collimator and penumbra. Sometimes patients needed to flex their knees to encompass the dosimetric field. To improve the uniformity of dose distribution along the long axis of the patients within ± 10%, the empty volume between the patients and custom-made couch lucite walls was filled with bags of rice (Fig. 1). It has been observed that rice exhibits the same attenuation characteristics as human tissue, and provides uniform thickness, thereby minimizing absorption variations and ensuring acceptable dose uniformity. Additionally, it acts as immobilization for the patient and simplifies the physical dosimetry.

A bilateral, parallel opposed field technique with 6 MV photon energy was used, and the couch was rotated 180 degrees between the treatments. The prescribed dose for every patient was 3 Gy in a complete session, delivered to the mid-depth of the patient at the level of the umbilicus.

### Radiation beam characteristics

Shielding was not needed due to the low total dose. Beam profiles, percentage depth dose (PDD), and output calibration of the linear accelerator at the treatment position were measured before each TBI treatment. These measurements were necessary because the parameters used to characterize the X-ray beam at the standard treatment distance of 100 cm may not be valid for extended treatment.

A manual monitor unit (MU) calculation was done based on the data obtained from the phantom measurement in the extended SSD condition. For the patient with a lateral shift less than equal to 44 cm, the total MUs calculated was 6082, and for the patient with a lateral shift less than equal to 52 cm, it was 7140 MU. The dose rate for each treatment is 600 MU/min. Care was taken to avoid any metal objects on the patient's body that may perturb the dose distribution.

### Statistical analysis

Patients were followed up closely during inpatient visits and monthly on an outpatient basis. Demographic data, treatment details, and major toxicities were analyzed with appropriate descriptive statistics.

### Ethical consideration

This retrospective study by chart review was approved by the Nepal Health Research Council to analyze the results of HSCT using TBI as a conditioning regimen along with chemotherapy.

## Results

### Patients and transplantation characteristics

**Fig. 2:**
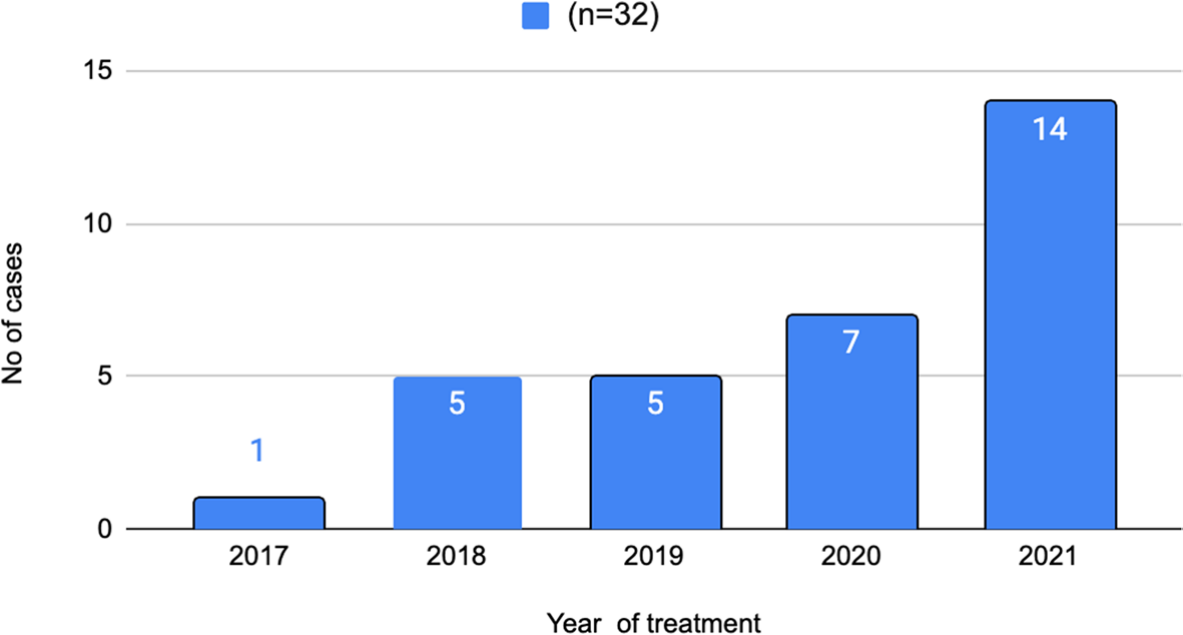
Number of patients undergoing total body irradiation

The TBI program started in 2017, and 32 patients have been treated so far. The number of patients has increased steadily each year (Fig. 2). Patients’ demographics are shown in Table 1. The most common diagnoses were aplastic anemia (*n* = 15) and Acute Myeloid Leukemia (AML) (*n* = 10). The most common conditioning regimen was fludarabine and cyclophosphamide in all patients, while ATG was added to the conditioning regimen in 2 patients.

**Table 1:** Patient’s and treatment characteristics (*n* = 32)

Variables	Median (range) or frequency	Percentage
Age	20.5 (5–48) years	
Sex		
Male	20	62.5%
Female	12	37.5%
Diagnosis		
Aplastic anemia	15	46.8%
AML	10	31.25%
ALL	3	9.3%
Others	4	12.5%
Conditioning regimen		
Fludarabine + cyclophosphamide	29	90.6%
Fludarabine − cytarabine ATG	2	6.2%
Busulfan + fludarabine	1	3.1%
TBI dose		
2 Gy	4	12.5%
3 Gy	28	87.5%

*AML* acute myeloid leukemia, *ALL* acute lymphocytic leukemia, *ATG* anti-thymocyte globulin, *TBI* total body irradiation, *Gy* Gray

### Post-transplant outcome

Engraftment was achieved in all patients. Out of the first four consecutive patients, two developed secondary graft failure due to cytomegalovirus (CMV) infection. Both patients were heavily pretreated in the past. The first patient who developed secondary graft failure was diagnosed with relapsed, refractory acute lymphoblastic leukemia (ALL) and received chimeric antigen receptor (CAR)-T cell therapy. Similarly, the second patient was heavily pretreated for relapsed refractory Hodgkin’s lymphoma. Starting with the fifth patient, the TBI dose was escalated from 2 to 3 Gy, and there were no subsequent graft failures.

The major non-infectious complications observed were acute and chronic GVHD, hemorrhagic myocarditis, and hepatic veno-occlusive disease.

Infectious complications in the early post-transplant period were hepatic candidiasis (*n* = 1) and CMV infection (*n* = 11) with CMV-induced secondary graft failure in two patients (Table 2). None of the patients in the study developed lung fibrosis, hypothyroidism, cataract, secondary malignancy, or heart failure.

**Table 2:** Complications during BMT

Complications	Number	Percentage
Acute GVHD	5	15.6%
Chronic GVHD	3	9.3%
CMV infection	11	34.3%
CMV induced secondary graft failure	2	6.2%
Hemorrhagic myocarditis	1	3.1%
Hepatic candidiasis	1	3.1%
Veno occlusive disease	3	9.4%
Grand total (The number may not add up to 100)	26	100

*GVHD* graft-versus-host disease, *CMV* cytomegalovirus

### Survival

**Fig. 3:**
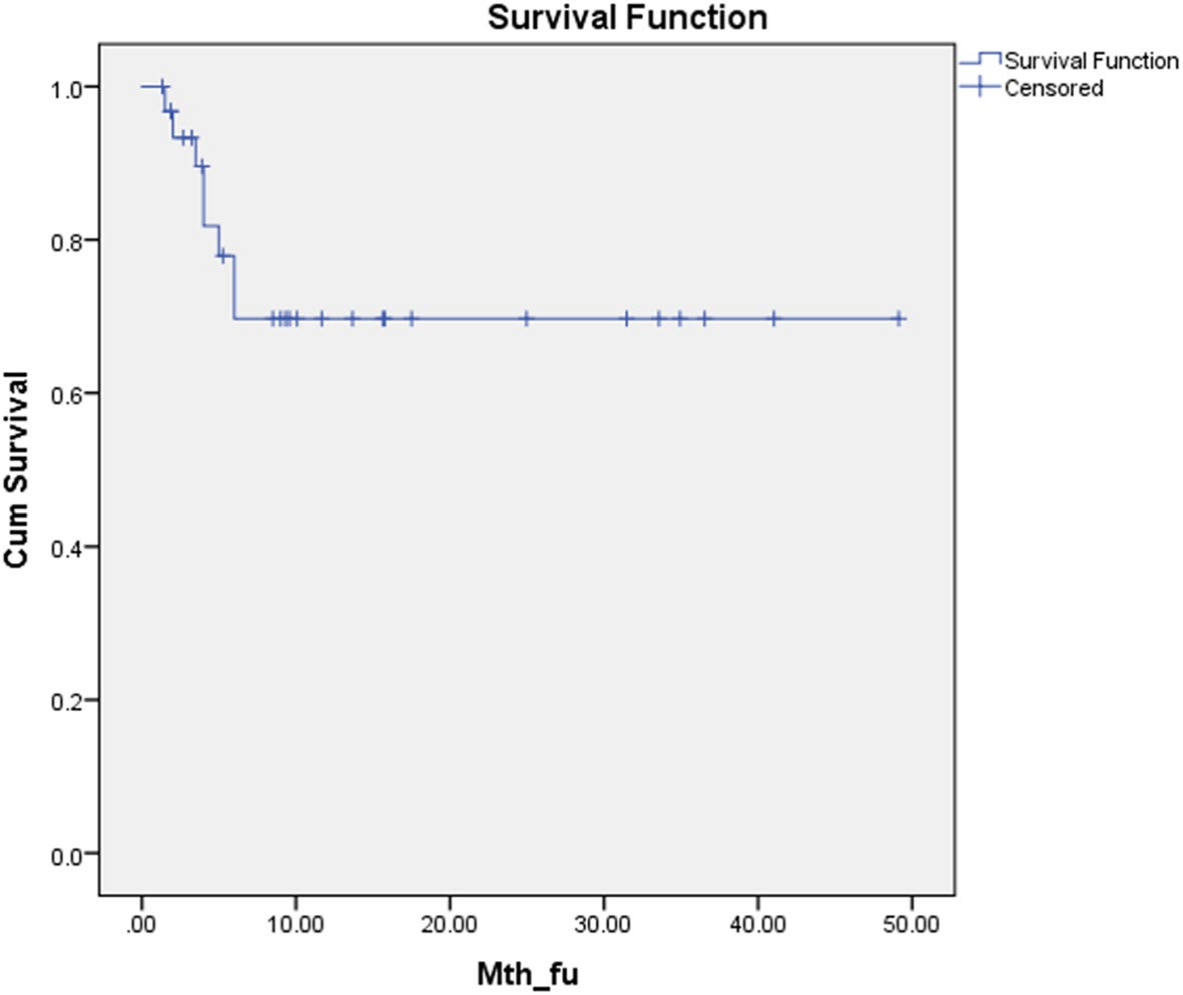
Kalpan-Meier survival curve of study population

The median follow-up was 22 months. At the last follow-up, 24 of 32 patients (75%) were alive, 7 (21.9%) were dead, and one was lost to follow-up. The 1-year overall survival was 70% (Fig. 3). Causes of death were CMV-induced secondary graft failure (*n* = 2), hepatic veno-occlusive disease (*n* = 3), hemorrhagic myocarditis (*n* = 1), and one patient who developed chronic renal GVHD.

## Discussion

In this report, we have described the technical aspects and results of our TBI program. Our main challenge was to start the first TBI program in the country in coordination with two different centers which has not yet been reported in the literature.

The TBI patient was taken as the first case before routine treatment time to start routine radiotherapy at our regular time. This did not interfere with routine radiotherapy treatment on a linear accelerator, which is important in centers with busy schedules. Initially, the TBI treatment took around one to 2 h of linear accelerator (LINAC) time, but now with more experience, it is routinely done within a 30-min slot.

The total treatment time from the bone marrow transplant suite to the back for the first few cases was 3.5–4 h, but with the streamlining of the process and the development of standard operating procedures, this has now been reduced to below 2 h.

The most challenging part of this type of treatment, which involves resources from different centers, is coordination between different teams. Using a free instant messaging app (Viber) in our case) is an easy, cost-effective, and real-time solution that needs to be expanded in medical sectors requiring coordination [8]. The confidentiality of the patient’s information was not harmed, and they were satisfied with the use of free-to-use apps like Viber. Earlier studies have demonstrated the use of such apps in medical education, improving the healthcare delivery system in developing countries [9, 10].

In our study, we showed that complicated treatments such as HSCT can be performed when good coordination between two physically distant centers is established. Patients undergoing hematological malignancies are more likely to experience episodic neutropenia, which is often associated with an increased risk of bacterial infections, particularly in recipients of allogeneic grafts with prior myeloablative conditioning regimens [11]. However, none of the patients were defined as having developed infections like sepsis or fungal pneumonia in our case. We believe this was possible because we used a reduced conditioning regimen, and apart from the aplastic anemia patients, most of the other patients were not severely neutropenic on the day of the transfer to the radiotherapy center.

GVHD remains the most deliberative adverse event after HSCT. In our study, acute GVHD and chronic GVHD were present in 5 and 4 patients, respectively. The use of high-dose post-transplant cyclophosphamide may have resulted in a low incidence of GVHD in our cohort [12]. However, post-transplant cyclophosphamide therapy has been shown to increase the rate of cytomegalovirus infection [13]. In our cohort, cytomegalovirus infection was seen in 11 patients (34.3%).

The complication rate in our series is comparable to other studies in similar populations [14, 15]. Our particular concern was infectious complications, the rates of which are similar to those reported in the literature [16, 17]. None of our patients developed invasive fungal infections. Although secondary malignancy is a concern in TBI-based regimens, the incidence in the low-dose TBI-based protocol is comparable to that of myeloablative chemotherapy alone, and none of our patients developed a second malignancy in the study period [18].

Guidelines for establishing HSCT in low-income countries suggested avoiding TBI in blood and marrow transplant (BMT) programs due to the cost of adding radiotherapy facilities to the BMT program [19]. However, TBI is a necessary part of certain preparative regimens, and our approach allows us to use those regimens without significantly increasing the program’s cost. The cost of TBI in our setting was USD 250 which is very economical. The cost calculation for radiotherapy treatment encompassed direct treatment, equipment (purchase, ROI, maintenance), overhead, infrastructure, and patient-related costs.

The costs associated with the diagnosis and treatment of hematological disorders present enormous financial toxicity to patients [20]. The average cost of an allogeneic stem cell transplant in our cohort was USD 13,000 [21]. This is still high when compared with our gross domestic product (GDP), but extraordinarily low compared to the West and comparable to that of neighboring countries [22]. We presume that this low cost was possible largely because of the use of generic medication during conditioning chemotherapy and partly due to the use of TBI in the conditioning regimen.

Our study has several limitations. First, this study consisted of a small number of cases. Secondly, there were some variations in the conditioning regimen; therefore, it is difficult to determine the cause-effect relationship between TBI and a particular adverse effect.

## Conclusions

In conclusion, this study explored the feasibility of conducting TBI-based stem cell transplant programs in centers that lack in-house facilities for radiotherapy without compromising the patient’s outcome. This is relevant to many centers in developing countries that have facilities for hematology services but can not offer TBI-based transplants due to a lack of in-house radiotherapy services.

## Data Availability

The data that support the findings of this study are not publicly available due to sensitivity and privacy concerns of the patient. However, data can be made available from the corresponding author upon reasonable request. Data are also available from the authors and with permission of the corresponding author, which are located in controlled access data storage system at Kathmandu Cancer Center.

## Abbreviations

ALL: Acute lymphoblastic leukemia
AML: Acute myeloid leukemia
BMT: Blood and marrow transplant
CAR: Chimeric antigen receptor
CMV: Cytomegalovirus
GDP: Gross domestic product
GVHD: Graft-versus-host-disease
Gy: Gray
HLA: Human leukocyte antigens
HSCT: Hematopoietic stem cell transplant
LINAC: Linear accelerator
LMI: Low- and middle-income country
MU: Monitor units
PDD: Percentage depth dose
SSD: Source-to-skin distance
TBI: Total body irradiation
